# Mobile signaling big data, healthcare-seeking flows, and multidimensional healthcare access in Shanghai: implications for healthcare supply–demand matching and medical security governance

**DOI:** 10.3389/fpubh.2026.1841112

**Published:** 2026-06-02

**Authors:** Qing Guo, Hengna Ren, Xinmiao Shao, Wenxiao Yang

**Affiliations:** 1 Business School, University of Shanghai for Science and Technology, Shanghai, China; 2 School of Marxism, Shanghai University of Engineering Science, Shanghai, China

**Keywords:** healthcare supply–demand matching, healthcare-seeking flows, mobile signaling data, multidimensional healthcare access, Shanghai, tiered hospital system

## Abstract

**Background/purpose:**

Broad medical insurance coverage alone does not ensure that a multilevel medical security system works effectively. In a megacity, the key issue is whether residents can reach suitable care, keep choices across provider tiers, and use high-quality services within daily time–space constraints. Proximity-based measures often miss this problem because they do not capture cross-district healthcare-seeking, preferences for higher-level hospitals, or the concentration of quality resources in limited urban locations. Using Shanghai as a case, this study used Mobile signaling big data to identify spatial inequalities in healthcare access and to inform service-side improvement.

**Methods:**

This study used anonymized mobile signaling records from March 2019 to reconstruct a directed, weighted healthcare-seeking flow network linking demand units with 352 hospitals in Shanghai. This study then constructed the Multidimensional Healthcare Access Capability Index (MHACI) with entropy-weighted TOPSIS. The index integrated spatial accessibility, healthcare-seeking choice diversity, and connectivity to high-quality healthcare resources. Multiscale geographically weighted regression (MGWR) was used to estimate associations of MHACI with demographic structure, economic capacity, transport infrastructure, population density, and local healthcare capacity across space.

**Results:**

Healthcare-seeking flows in Shanghai were concentrated toward the urban core, with cross-district movement. MHACI showed a clear center-periphery gradient. Aging share was positively related to MHACI mainly in central and adjacent areas, whereas child share was linked to lower MHACI in core districts. Per capita GDP and local healthcare capacity had positive but localized effects. Population density shifted from a crowding burden in central areas to an agglomeration advantage in peripheral areas, while transport infrastructure mainly acted as a background condition.

**Conclusion:**

Unequal healthcare access in Shanghai reflects a mismatch between fixed healthcare provision and healthcare-seeking flows across urban space. Improving a multilevel medical security system requires not only formal coverage, but also stronger primary care, better connectivity across provider tiers, and place-specific governance in core, peri-urban, and outer suburban areas.

## 1 Introduction

Optimizing healthcare resource allocation and improving spatial equity remain central concerns in public health governance and health geography (1, 2). Broad medical insurance coverage, however, does not by itself ensure effective healthcare access in practice. As cities expand, unequal access to healthcare has become an increasingly important constraint on population health (3, 4). Current literature still struggles to capture realized healthcare access when healthcare-seeking mobility is directly observed. Many studies continue to rely on static indicators such as hospital bed supply, population size, and geographic distance (5, 6). In practice, this reduces healthcare access to a question of proximity. In tiered hospital systems, healthcare seeking is rarely a simple move to the nearest provider. It is shaped by hierarchy, competition, and the rhythms of everyday mobility (7, 8). At the same time, expanding transport systems and rising job-housing separation have widened the spatial range of daily activities (9, 10). As daily activity spaces expand, observed healthcare-seeking flows increasingly cross administrative and neighborhood boundaries, weakening the long-used assumption of nearby healthcare seeking. One-dimensional measures, convenient as they may be, no longer capture the supply–demand tensions behind spatial inequity.

Recent advances in mobile signaling data, together with electronic medical records and other digital traces, have opened new possibilities for addressing these limitations (11, 12). To respond to this gap, this study introduces the Multidimensional Healthcare Access Capability Index (MHACI) to move beyond distance alone. MHACI treats healthcare access as a joint outcome of spatial reachability, behavioral choice, and network connectivity. It asks whether residents can reach healthcare resources under spatial impedance, whether they retain meaningful choice within the healthcare-seeking flow network, and how well their communities are connected to high-quality hospitals.

Taken together, these dimensions provide a more realistic account of actual healthcare access capability. Mapping MHACI merely initiates the inquiry. It is equally important to explain why multidimensional healthcare access varies across demand units and how different urban conditions shape that variation. Existing studies have linked healthcare access to socioeconomic and geographic conditions, but many still rely on global models such as ordinary least squares (OLS), which can mask local differences in complex urban settings (13, 14). Geographically weighted regression (GWR) addresses part of this problem by allowing coefficients to vary across space, yet it still assumes that all explanatory variables operate over a common spatial scale (15–17). In megacities, however, the drivers of healthcare access do not operate at a single spatial scale. Demand pressure, local supply capacity, economic conditions, and transport infrastructure may shape healthcare access over different geographic ranges. For this reason, multiscale geographically weighted regression (MGWR) is used to estimate how urban factors are associated with MHACI across variable-specific spatial scales.

Shanghai, China, serves as the empirical case. The analysis follows a stepwise framework linking network construction, index development, and multiscale spatial analysis. First, residents’ healthcare-seeking trajectories are identified from mobile signaling data to construct a directed and weighted healthcare-seeking flow network. Next, geographic information and network measures are combined to assess MHACI and map its spatial pattern. Finally, urban factors related to geography, socioeconomic conditions, and public health are incorporated into the MGWR model to examine their spatial associations with MHACI. In this way, the study moves from healthcare-seeking flows to multidimensional healthcare access, and from spatial inequality to questions of healthcare supply–demand matching within Shanghai’s tiered hospital system. The findings can inform healthcare resource allocation and supply–demand matching within Shanghai’s tiered hospital system. They may also provide empirical insights for other large cities facing similar tensions among hierarchical healthcare provision, mobility-driven demand, and uneven access.

## 2 Study area, data, and healthcare-seeking flows

### 2.1 Study area and overview

**Figure 1:**
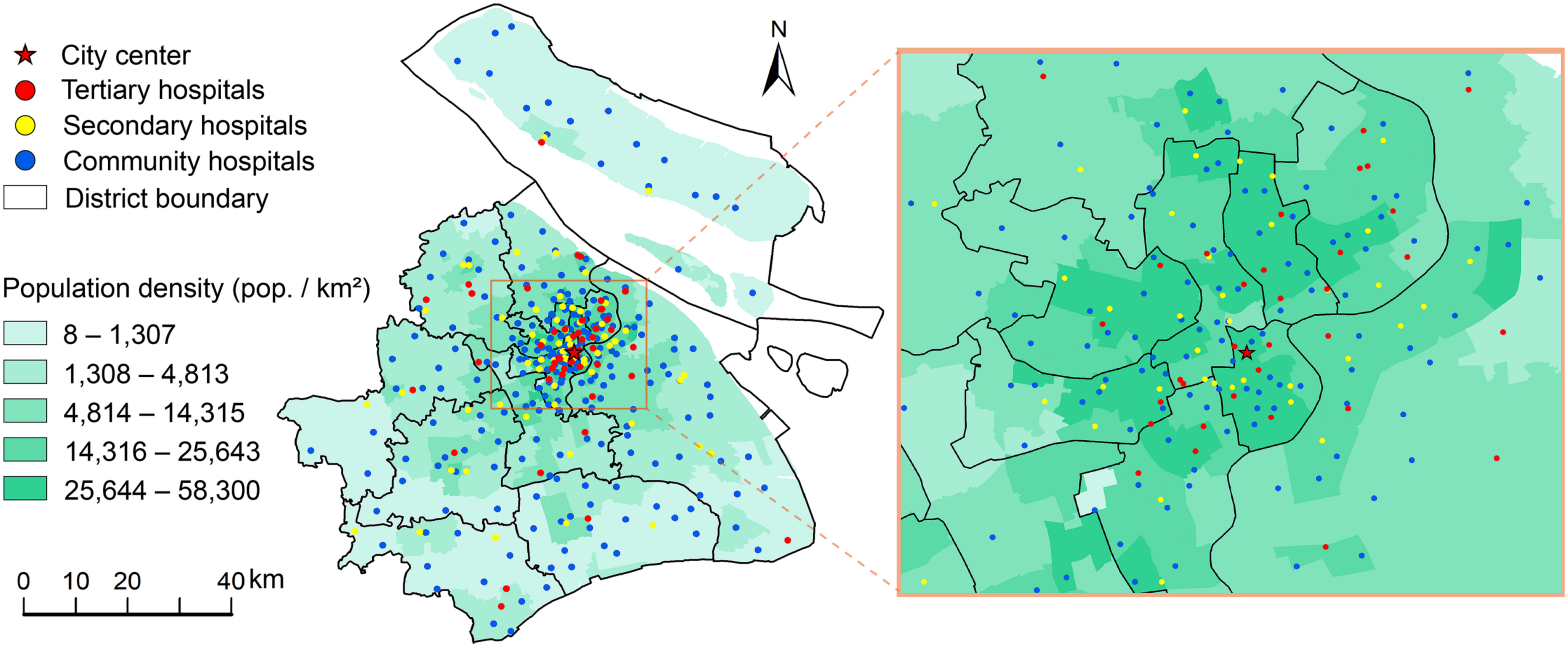
Spatial distribution of population and public general hospitals in Shanghai.

As a highly developed metropolis with 16 administrative districts, Shanghai displays a polycentric spatial structure composed of an urban core and peripheral new towns. Figure 1 elucidates this demographic reality. With continued urban expansion, the city has experienced pronounced job-housing separation (18). Stemming from decades of historical path dependence, the local healthcare architecture reveals a striking hierarchy: elite tertiary institutions remain rigidly sequestered within the central city, leaving the expansive peri-urban reaches heavily reliant on lower-tier secondary and community facilities (19). Such spatial mismatch breeds profound disparity. Compounded by this centripetal hoarding of premium care, the geographic maldistribution fundamentally entrenches inequities in realized healthcare access (20). Framed by these intense mobility rhythms and a rigidly stratified medical supply, this megacity emerges as an unparalleled laboratory for dissecting spatial justice in public health. To strictly align the analytical timeline, all demographic, economic, and institutional baselines capture the 2019 annual cross-section; parallel to this, the high-resolution mobility trajectories are anchored specifically to March 2019.

### 2.2 Data acquisition and processing

#### 2.2.1 Healthcare facility dataset

Serving as the core supply units in the healthcare-seeking flow network, public general hospitals were deliberately targeted as the main facilities through which residents seek healthcare. Institutional realities dictate this alignment. Across the Chinese metropolitan fabric, public general hospitals maintain an absolute dominance over medical provision, capturing broad spatial coverage and a wide service scope (21). Driven by the necessity to map everyday healthcare seeking behavior under common medical needs, specialty hospitals were purposefully excluded to filter out behavioral noise from highly specific population groups and less common disease patterns. These mainstream institutions also contain most high-quality healthcare resources. They yield a highly robust observational basis for examining both the spatial distribution of healthcare provision and the micro-level patterns of healthcare-seeking flows.

To formulate a high-fidelity spatial inventory, hospital names, addresses, and levels were systematically extracted directly from official lists issued by the Shanghai Municipal Government (22–24). Rigorous spatial triangulation followed. Harvested from a public geographic information platform, hospital point-of-interest (POI) data underwent a painstaking manual reconciliation against these official rosters to ensure consistency in facility identification and spatial location (25). The final sample ultimately crystallized around 352 hospitals, comprising 48 tertiary hospitals, 62 secondary hospitals, and 242 community hospitals. Beyond mere coordinates, additional information for 2019 covering approved bed numbers, numbers of licensed physicians, fiscal budgets, and building area at different hospital levels underwent meticulous synthesis via statistical yearbooks, official websites, and offline verification (26, 27).

#### 2.2.2 Geographic and socioeconomic data

Vector boundary base maps for Shanghai and its administrative divisions were extracted from the National Catalogue Service for Geographic Information Resources (28). Context demands human metrics. Detailing permanent residents and gross domestic product (GDP), key socioeconomic data underwent synthesis via the population census, the Shanghai Statistical Yearbook, and localized statistical bulletins (29). Parallel to this, data for the urban transport network were harvested through an open-source mapping platform (30). Rigorous alignment followed. To guarantee accuracy in spatial calculations and spatiotemporal consistency, all spatial data were transformed into a common projected coordinate system. The year 2019 anchors the empirical framework.

#### 2.2.3 Mobile signaling data

Unveiling the actual mobility dynamics across the metropolis, our empirical framework utilizes a continuous one-month dataset of mobile phone signaling logs secured from a major telecommunications operator in Shanghai. Temporally, the observation encompasses all of March 2019. Within this database, extracted records fuse spatial coordinates with hourly timestamps to yield the base-station-level locational pings essential for reconstructing complex daily trajectory sequences and stationary periods. Ethical compliance dictated all subsequent processing. Prior to analytical access, irreversible scrambling and aggregation systematically eradicated all user identifiers, guaranteeing a final analytical sample entirely devoid of personal demographic information.

The construction of the healthcare-seeking flow network and the calculation of related indicators drew on multiple spatial, statistical, and mobility datasets. A detailed summary of data sources, temporal references, original scales, and analytical use is provided in Supplementary Table. The study was anchored to the 2019 research period. Hospital attributes, population, and socioeconomic variables were aligned to the 2019 annual cross-section, while mobile signaling records covered March 2019. Transport infrastructure and administrative boundary data were used as spatial reference layers and were matched to the study setting as closely as possible. All spatial datasets were projected into a common coordinate system. Variables from different original spatial units were linked to the 352 demand units or 352 hospitals according to their analytical roles.

### 2.3 Healthcare-seeking trajectory identification and flow network construction

#### 2.3.1 Screening of mobile signaling data

Data screening began with the extraction of fields required for analysis, followed by the removal of duplicate entries and noisy records. Frequent switching between adjacent base stations within a short period was consolidated into a single stay point so that false movement would not be overstated. Records indicating implausible spatial displacement over a limited time interval were also discarded. Together, these procedures reduced positioning errors associated with abnormal signals (31).

To preserve stable and representative healthcare-seeking patterns, the subsequent analysis was restricted to Shanghai’s resident sample. An active day was defined as any calendar day with at least one valid geographic ping within the municipal boundary. A user entered the resident sample only when more than 15 active days were recorded during the observation month; all others were treated as transient populations or short-term visitors. Hereafter, the retained sample is referred to as residents.

#### 2.3.2 Identification of workplace and home locations

Home and workplace locations were inferred from repeated stay patterns within predefined temporal windows by means of DBSCAN clustering (32, 33). For workplace identification, the observation window covered 09:00–11:00 and 13:00–17:00 from Monday to Friday. Spatiotemporal records falling within these periods were used to generate daytime clusters. A cluster was retained as a workplace only if it appeared on at least five working days and accounted for more than 50% of the user’s active working days during the month. The geometric center of the retained cluster was then taken as the workplace location.

Home location was determined from nighttime activity. The relevant window extended from 22:00 to 06:00 on the following day, and stay records within this period were grouped using the same clustering procedure. A cluster was treated as a home location when it was observed on at least five nights and covered more than 50% of the user’s total nighttime active days during the observation period. Once these conditions were met, the geometric center of that cluster was used as the home location coordinates.

#### 2.3.3 Identification of healthcare-seeking trajectories

Noise removal. To reduce misclassification of work-related hospital presence, such as that of hospital staff, and hospitalization-related stays as healthcare-seeking behavior, a 100 m buffer was applied around hospital buildings. The buffer allowed for the positional uncertainty of mobile signaling records and the spatial extent of hospital campuses in dense urban settings. Users whose home or workplace coordinates fell within this area were excluded. This step was used for noise removal only. A hospital visit was retained only when the record matched a hospital building footprint during outpatient peak hours and the stay lasted longer than 1 h.

Hospital visits and destination identification. For the remaining users, trajectory records during daily outpatient peak hours (08:00–11:00 and 13:00–16:00) were spatiotemporally matched to hospital locations. Rather than treating all hospital presence as a visit, we retained only those stays located within a hospital building footprint that lasted longer than 1 h. The specific hospital associated with such a stay was then assigned as the flow destination. These time windows and duration thresholds were instrumental in filtering out brief, transient noise. Given our focus on general healthcare-seeking behavior, nighttime emergency visits and hospitalization-related stays were not included in the final flow dataset.

Delineation of healthcare-seeking demand units. Voronoi-based healthcare-seeking demand units were constructed with hospital point locations as generators and treated as the origin units of healthcare demand. This design has two advantages. It ensures mutual exclusivity and complete spatial coverage (34), and the one-to-one correspondence between hospitals and demand units makes it possible to distinguish clearly between origins and destinations in the bipartite network. Voronoi units were used to assign home and workplace locations to origin units. They were not treated as hospital catchment areas, and no assumption was made that residents were evenly distributed or sought care nearby. Destination hospitals were identified from mobile signaling trajectories.

Origin identification. For each valid healthcare visit made by a permanent resident, the trajectory was traced backward to the most recent workplace or home location recorded before the hospital visit. That location was then assigned to the corresponding healthcare-seeking demand unit through point-in-polygon matching and used as the origin of the flow. To capture the primary healthcare choice and avoid duplicate counting, only the first healthcare-seeking path of each resident on each day was retained. Subsequent referrals or trajectories involving multiple stop points were excluded, and each retained path was treated as a single-hop path. The Voronoi-based healthcare-seeking demand units in this study function only as spatial containers for home and workplace locations and their associated attribute variables. They do not impose an assumption of nearby healthcare seeking; the observed healthcare-seeking flows are identified directly from mobile signaling data.

#### 2.3.4 Formal specification of the healthcare-seeking flow network

**Figure 2:**
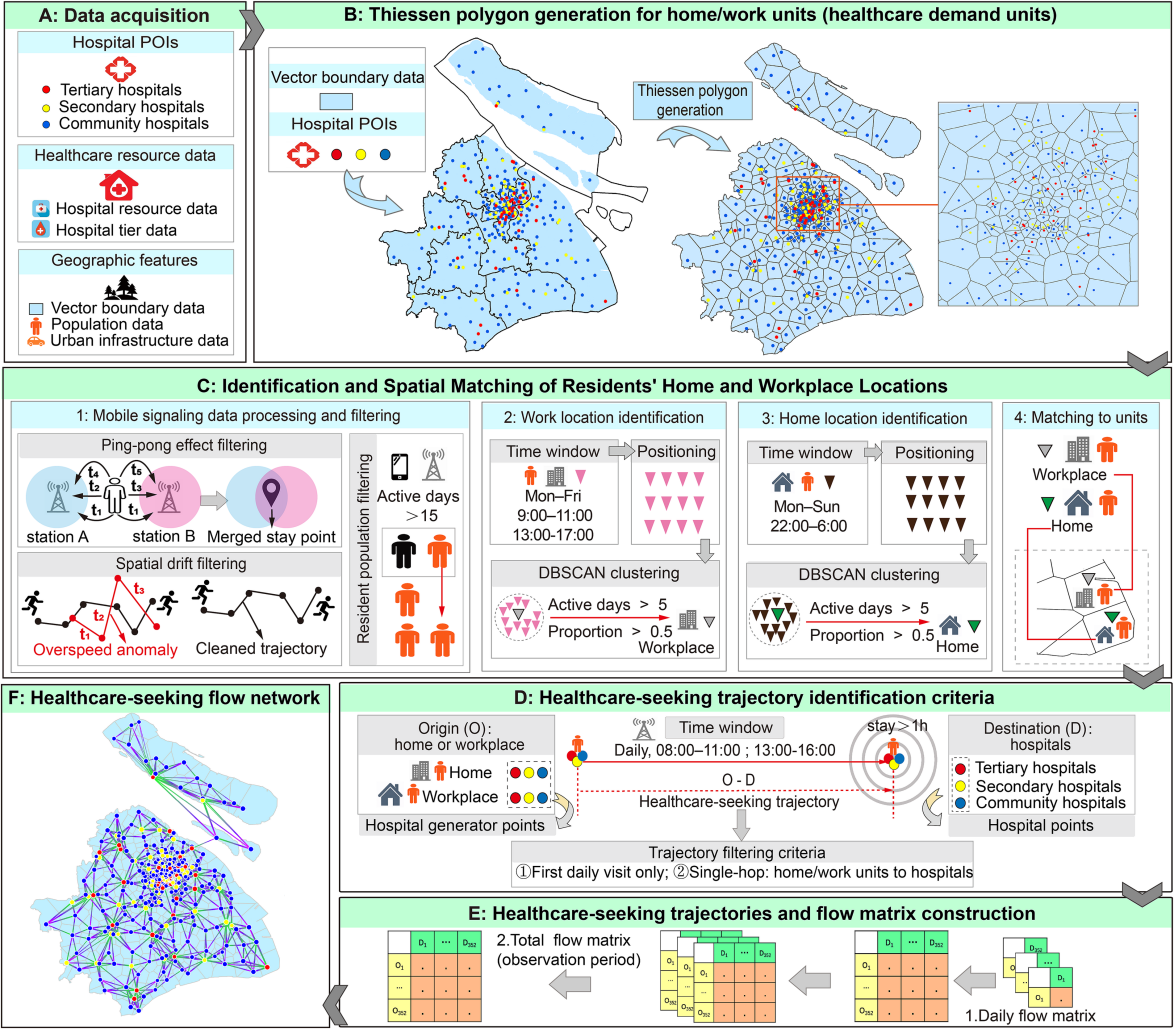
Construction steps of the healthcare-seeking flow network. **(A)** Data acquisition; **(B)** Thiessen polygon generation for home/work units (healthcare demand units); **(C)** Identification and spatial matching of residents’ home and workplace locations; **(D)** Healthcare-seeking trajectory identification criteria; **(E)** Healthcare-seeking trajectories and flow matrix construction; **(F)** Healthcare-seeking flow network.

Figure 2 summarizes the procedure used to build the healthcare-seeking flow network. Following this framework, a directed and weighted network for Shanghai was represented as G=(O,D,E,W) . In this representation, $O=\{O_{i}\}$ denotes the Voronoi-based healthcare-seeking demand units generated from the point locations of public general hospitals in Shanghai (hereafter referred to as demand units). $D=\{D_{j}\}$ denotes the public general hospitals that function as healthcare supply units. $E=\{e_{\mathrm{ij}}\}$ represents healthcare-seeking paths, where $e_{\mathrm{ij}}$ links demand unit $O_{i}$ to hospital $D_{j}$ . $W=\{w_{\mathrm{ij}}\}$ records the corresponding monthly cumulative healthcare visits from $O_{i}$ to $D_{j}$ in March 2019. To represent the movement cost embedded in these flows, a spatial impedance matrix $R=\{r_{\mathrm{ij}}\}$ was constructed. Under a parsimonious specification, adjusted distance was used as the proxy for spatial impedance. Within each demand unit $O_{i}$ , Monte Carlo simulation was used to generate 2,000 sample points. The Euclidean distance from each point to hospital $D_{j}$ was then calculated, and the arithmetic mean of these distances was taken as the straight-line distance $d_{\mathrm{ij}}$ between $O_{i}$ and $D_{j}$ . Because straight-line distance tends to underestimate actual travel under road detours and river barriers, $d_{\mathrm{ij}}$ was further corrected by a nonlinearity coefficient α (35). For general land routes, α was set at 1.3. For cross-river paths to and from Chongming Island, α was set at 1.8 to reflect the additional detour cost. Spatial impedance was thus defined as $r_{\mathrm{ij}}=d_{\mathrm{ij}}\times \alpha$ , and the resulting values formed the matrix R . This measure was used as a transparent and reproducible proxy for spatial impedance, rather than as a direct estimate of travel time or travel cost.

## 3 Measurement framework and model specification

### 3.1 Construction of the multidimensional healthcare access capability index (MHACI)

Conventional measures of healthcare accessibility rely heavily on distance-decay assumptions. In complex urban settings, however, such measures are not sufficient to capture whether residents can achieve stable, equitable, and meaningful access to healthcare under shifting patterns of demand and mobility. To address this limitation, this study evaluates healthcare access capability within each demand unit through three interrelated dimensions: geographic space, individual behavior, and network structure. The Multidimensional Healthcare Access Capability Index (MHACI) integrates spatial accessibility, healthcare-seeking choice diversity, and connectivity to high-quality healthcare resources. Together, these dimensions provide a fuller basis for assessing how access is actually realized across the city and for identifying the spatial inequities that matter for sustainable urban health governance.

#### 3.1.1 Spatial accessibility

This indicator measures the spatial potential of residents in $O_{i}$ to overcome geographic distance and reach healthcare resources at different levels. Equation 1 defines this indicator. In the equation, $q_{j}$ denotes the hierarchical weight of destination hospital $D_{j}$ , where tertiary hospitals are assigned a value of 3, secondary hospitals a value of 2, and community hospitals a value of 1. This tier weight reflects the functional hierarchy of hospitals in Shanghai. It avoids treating community, secondary, and tertiary hospitals as equivalent destinations. $r_{\mathrm{ij}}$ is the adjusted spatial distance; and the distance-decay parameter β is set to 1 (36). A larger value of $A_{i}$ indicates a lower distance threshold for accessing healthcare resources and, therefore, stronger spatial accessibility for residents in $O_{i}$ .


$$A_{i}=\sum_{j\in D}\frac{q_{j}}{r_{\mathrm{ij}}^{\beta }}$$ (1)

#### 3.1.2 Healthcare-seeking choice diversity

This indicator is used to assess the diversity of healthcare choices available to residents in $O_{i}$ within the observed healthcare-seeking flow network. Its calculation is shown in Equation 2. Here, $p_{\mathrm{ij}}$ = $w_{\mathrm{ij}}/\sum_{k}w_{\mathrm{ik}}$ represents the probability that residents in $O_{i}$ seek healthcare at $D_{j}$ . A higher value of $E_{i}$ indicates a more diversified set of healthcare destinations and stronger resilience in healthcare seeking for residents in $O_{i}$ .


$$E_{i}={\left(\sum_{j\in D}p_{\mathrm{ij}}^{2}\right)}^{-1}$$ (2)

#### 3.1.3 Connectivity to high-quality healthcare resources

High-quality healthcare resources are further examined from the perspective of network connectivity by applying the HITS algorithm (37, 38). A choice probability matrix $P=[p_{\mathrm{ij}}]$ is first constructed from observed healthcare-seeking flows in order to reflect residents’ actual healthcare preferences. Bidirectional iterative updating is then carried out. As shown in Equation 3, the algorithm identifies highly attended core hospitals in the network through authority scores $a^{\left(t\right)}$ and evaluates how effectively each unit connects to these high-quality resources through hub scores $h^{\left(t\right)}$ . After convergence, the resulting score for demand unit $O_{i}$ is recorded as $C_{i}$ . A larger $C_{i}$ indicates stronger network-based access to high-quality healthcare resources through the observed healthcare-seeking flows.


$$\begin{array}{c} a^{\left(t+1\right)}=P^{T}h^{\left(t\right)},\;h^{\left(t+1\right)}=Pa^{\left(t\right)} \end{array}$$ (3)

#### 3.1.4 Composite measurement of MHACI

The three dimensions were synthesized into the MHACI by means of entropy-weighted TOPSIS (39). Here, $A_{i}$ captures tier-weighted spatial accessibility, while $E_{i}$ and $C_{i}$ describe observed choice diversity and network-based connection to high-quality healthcare resources. MHACI therefore combines potential access to hospitals of different tiers with realized healthcare-seeking patterns. Rather than relying on preset weights, the contribution of each indicator was determined from its own information entropy, thereby minimizing subjective influence during the aggregation process. The relative closeness of residents in each $O_{i}$ to the ideal state of healthcare access is then calculated to obtain the MHACI for that demand unit. The calculation is shown in Equation 4, where $D_{i}^{+}$ and $D_{i}^{-}$ represent the distances from demand unit $O_{i}$ to the positive and negative ideal solutions, respectively. The closer MHACI is to 1, the stronger the overall healthcare access capability of residents in $O_{i}$ , and the weaker the spatial constraints they face in seeking healthcare.


$$\begin{array}{c} \text{MHAC}I_{i}=\frac{D_{i}^{-}}{D_{i}^{+}+D_{i}^{-}},\;\text{MHAC}I_{i}\in [0,1] \end{array}$$ (4)

### 3.2 Selection of explanatory variables

A clearer understanding of how urban factors shape spatial variation in MHACI across demand units is essential for identifying inequities in multidimensional healthcare access and for informing sustainable urban health governance. Drawing on the Andersen healthcare utilization model and theories from health geography, this study selects six explanatory variables across three domains: demand-side factors, enabling resources, and supply-side factors (40). To ensure spatial alignment in the subsequent modeling, all explanatory variables were measured or aggregated at the demand-unit level. Table 1 presents the definitions and descriptive statistics of all variables.

**Table 1:** Variable definitions and descriptive statistics.

Variable	Description	Mean	S. D.	Min	Max
MHACI	TOPSIS composite score	0.151	0.144	0.001	0.885
Aging share	Population aged ≥ 65 (share)	0.180	0.064	0.068	0.476
Child share	Population aged 0–15 (share)	0.093	0.017	0.031	0.139
Per capita GDP	GDP per capita (10,000 CNY / person)	16.306	7.979	5.580	39
Transport infrastructure	PCA index of bus stop, metro station, and road network densities	0.000	1.517	−2.953	4.151
Population density	Residents per square kilometer	13,155	11,752	145	54,379
Local healthcare capacity	PCA index of beds, personnel, departments, area, and budget	0.000	2.161	−3.539	5.906

Descriptive statistics are based on the modelling dataset. Transport infrastructure and local healthcare capacity are PCA-derived indices (mean ≈ 0). All explanatory variables were *z*-score standardized prior to model estimation.

#### 3.2.1 Demand-side variables

Demand-side variables reflect the level and structure of potential healthcare need. Older adults and children are typical high-need groups whose healthcare-seeking behaviors are especially sensitive to spatial constraints. Aging share, defined as the proportion of older adults in each demand unit, captures the frequent healthcare demand associated with chronic conditions. Because older adults often face physical decline, their healthcare seeking depends heavily on the physical accessibility and flexibility of local resources (41). Including this metric helps examine whether high-need aging populations fall into a pattern of spatial deprivation caused by resource mismatch. Child share, defined as the proportion of children in each demand unit, reflects a vulnerable form of healthcare demand highly dependent on accompaniment and caregiving. As young families increasingly concentrate in peripheral new towns while high-quality general hospitals remain clustered in the urban core, cross-district healthcare seeking imposes hidden spatiotemporal costs on these households (42). This variable evaluates how the spatial mismatch between outward demographic shifts and centripetal resource distribution contributes to inequities in healthcare access.

#### 3.2.2 Enabling resource variables

Enabling resources represent the conditions supporting residents’ mobility. Per capita GDP, measured at the demand-unit level, reflects local economic capacity, as higher-income groups are generally better able to absorb the financial and temporal costs of long-distance healthcare seeking (43). This metric tests whether regional economic advantage effectively translates into stronger multidimensional access capability. Transport infrastructure, measured via a PCA-based composite of metro station, bus stop, and road network density, reflects the physical conditions facilitating mobility (44). Strong transit networks compress spatiotemporal distance, lowering the geographic threshold for healthcare utilization. This variable assesses the policy buffering role of public transport in mitigating spatial inequities.

#### 3.2.3 Supply-side variables

Supply-side variables capture the local provision and competition environments faced by residents. Local healthcare capacity measures the overall service strength of the generator hospital associated with each demand unit. Because robust local supply can trigger a spatial lock-in effect that paradoxically constrains residents’ choice diversity (45), this indicator is included to gauge the degree to which local supply anchors overall access capability. Population density, measured at the demand-unit level, captures the dual spatial effects of resource agglomeration and healthcare crowding (46). It provides a direct assessment of how local resource advantages and competition interact across space to shape multidimensional healthcare access capability.

### 3.3 Model specification

Conventional ordinary least squares (OLS) models assume that the effects of explanatory variables remain constant across the study area. Geographically weighted regression (GWR) relaxes this assumption by allowing coefficients to vary with location, but it still requires all explanatory variables to operate over a single spatial scale (47, 48). In complex urban settings, however, different factors often vary unevenly across space, and the micro-level dynamics of healthcare-seeking flows can further amplify this spatial heterogeneity (49). Consequently, this study builds on the baseline model by introducing multiscale geographically weighted regression (MGWR), which allows each explanatory variable to adapt to its own optimal smoothing bandwidth and thus captures differentiated effects across spatial scales. The basic form of the model is given in Equation 5.


$$\begin{array}{c} \;y_{i}=\beta_{0}(u_{i},v_{i})+\sum_{m=1}^{k}\beta_{m}(u_{i},v_{i};bw_{m})x_{\mathrm{im}}+\varepsilon_{i} \end{array}$$ (5)

In this equation, $y_{i}$ denotes the MHACI score of demand unit $O_{i}$ ; $\left(u_{i},v_{i}\right)$ represents the geographic coordinates of the generator hospital associated with that unit; $x_{\mathrm{im}}$ is the value of the m -th explanatory variable; and $\varepsilon_{i}$ is the random error term. Unlike GWR, the local coefficient $\beta_{m}(u_{i},v_{i},bw_{m})$ is estimated on the basis of the variable-specific optimal bandwidth $bw_{m}$ .

To examine spatial heterogeneity in MHACI, the model was estimated with an adaptive bisquer kernel, which is better suited to the pronounced differences in area and density between urban and suburban spatial units. The optimal bandwidth for each variable was selected by minimizing the corrected Akaike information criterion (Amick) (50). All explanatory variables were standardized as z-scores before estimation to improve convergence stability and reduce scale-related interference. Local coefficients were evaluated against the adjusted critical t -values generated by the model, thereby limiting false significance from multiple testing.

Two diagnostic checks were carried out before model estimation. Multicollinearity was examined using the Variance Inflation Factor (VIF). Spatial dependence was then assessed with Global Moran’s *I* for both MHACI and the residuals of the OLS model. Given the clustering and mismatch often observed between urban healthcare provision and healthcare-seeking flows, this step was used to determine whether the global specification had adequately represented the underlying spatial pattern. If spatial autocorrelation remained in the OLS residuals, a multiscale framework would be more appropriate.

## 4 Results

### 4.1 Structure and characteristics of the healthcare-seeking flow network

**Figure 3:**
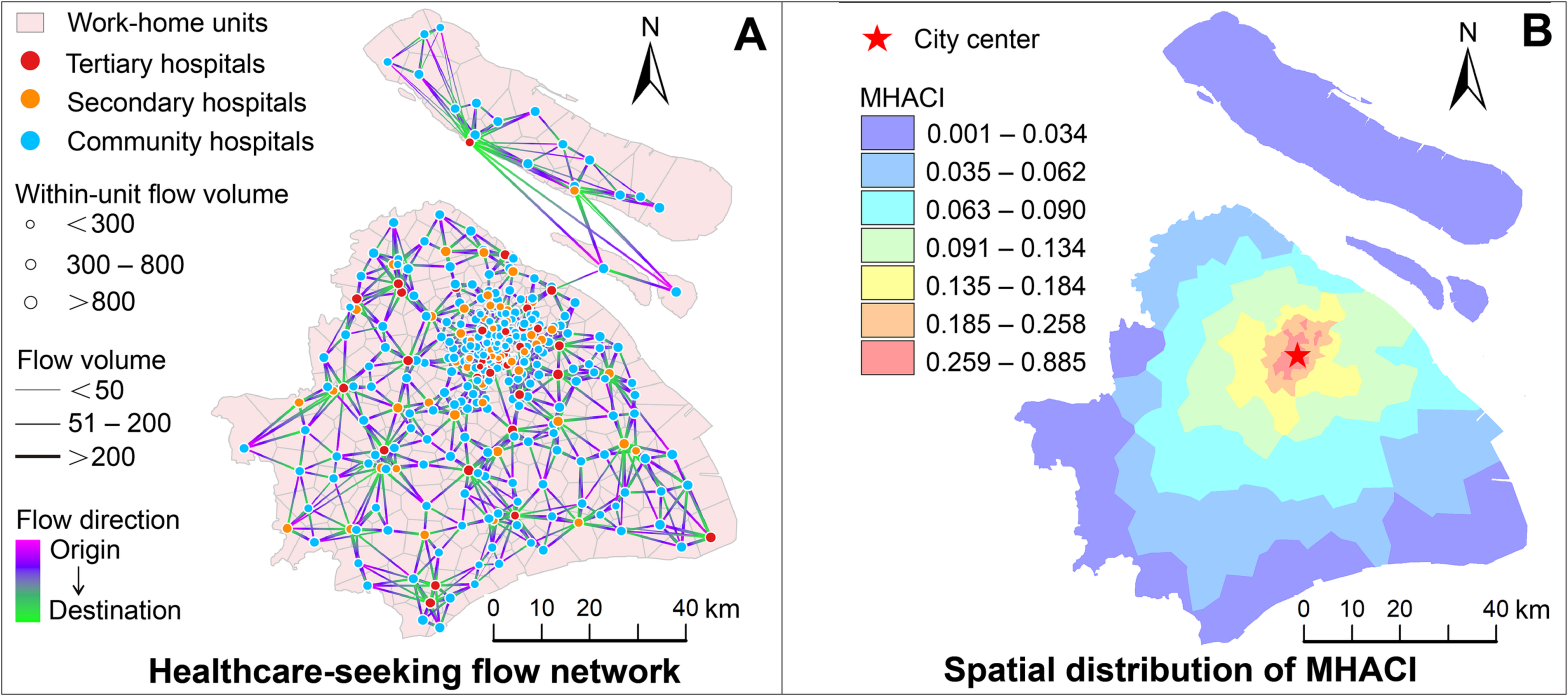
**(A)** Healthcare-seeking flow network in Shanghai for the top 5 outgoing nodes; **(B)** Spatial distribution of MHACI in Shanghai, classified into seven quantile-based classes.

A directed and weighted healthcare-seeking flow network was constructed with 352 demand units as origins and 352 hospitals as destinations (Figure 3A). During the observation period, 1,520,695 healthcare visits generated 17,122 valid healthcare-seeking paths, yielding a network density of 0.138 (Table 2).

**Table 2:** Descriptive statistics of the healthcare-seeking flow network.

Network attributes	Flow characteristic	Statistics
Network structure	Number of nodes	352
	Number of active edges ( $w_{\mathrm{ij}}\;$ > 5)	17,122
	Network density	0.138
Flow composition	Total healthcare-seeking flows ( $\sum w_{\mathrm{ij}}$ , monthly visits)	1,520,695
	Flow share: Tertiary / Secondary / Community	35.73% / 24.60% / 39.66%
	Top 10 hospitals’ share of total inflow	12.44%
	Within-unit flow share	12.47%
	Intra-district flow share	47.23%
	Cross-district flow share	40.30%
Spatial distance	Weighted average distance (km)	9.163

Within-unit flows link a demand unit to its corresponding hospital; intra-district flows remain within the same district; cross-district flows span different districts.

From the spatial distribution of network topology, healthcare-seeking flows in Shanghai are markedly denser and more frequent in the urban core, while the urban periphery shows a looser and more diffuse pattern (Figure 3A). As indicated by the legend, links with larger healthcare flows, shown as thick solid lines, converge on or toward high-level hospitals and the urban core. This pattern suggests a clear spatial concentration in healthcare-seeking mobility.

Across the full network, cross-district flows account for 40.30% of all healthcare visits, Intra-district flows for 47.23%, and Within-unit flows for 12.47% (Table 2). The weighted average travel distance of residents’ healthcare-seeking flows is 9.163 km. Figure 3A further reveals a substantial presence of long-distance trajectories. To capture spatial reality, the sheer dominance of cross-district flows and intra-district flows elucidates that residents’ healthcare-seeking behaviors frequently transcend immediate geographic constraints. Space rarely restricts choice. Beyond regional boundaries, the distribution of visits across hospital levels unveils another profound structural imbalance. Despite numbering only 48, tertiary hospitals command a massive 35.73% of total healthcare visits, contrasting sharply against the 62 secondary hospitals securing 24.60% and the 242 community hospitals absorbing the largest share at 39.66%. Extreme polarization dictates this network. Siphoning 12.44% of all visits citywide, a mere top 10 hospitals vividly underscore this severe inflow concentration (Table 2). Figure 3A directly corroborates this phenomenon. Marked as red nodes, high-level facilities aggressively attract dense inflow links portrayed by green directional lines. Through this visual evidence, these premium institutions unequivocally secure their central network position within the urban core, generating a powerful attraction for healthcare-seeking flows.

### 4.2 Spatial distribution of MHACI

Operationalizing the multidimensional healthcare access evaluation framework, the MHACI was mapped for residents across all 352 healthcare-seeking demand units in Shanghai. Spatial polarization defines this metropolis. Figure 3B elucidates a stark spatial hierarchy. Confined to a tier spanning 0.185 to 0.885, absolute capability remains sequestered within the high-density urban core, securing multidimensional healthcare access for inner-city populations. Beyond this central city, the index degrades precipitously to intermediate levels between 0.063 and 0.184. Parallel to this decay, scores plummet into a severe deficit tier bounded by 0.001 and 0.062 across isolated offshore island zones. Geography dictates vulnerability. Strikingly, this systematic outward attrition unveils profound spatial inequities, contrasting robust central capability against the glaring resource vacuums of the remote periphery. The Global Moran’s *I* value for MHACI was 0.827 ( p < 0.01), confirming significant spatial clustering and providing a statistical basis for the subsequent MGWR analysis.

### 4.3 Local effect estimation and spatial heterogeneity

Given the pronounced spatial clustering of MHACI, MGWR was used to examine how demand-side factors, enabling resources, and supply-side variables varied across space. Model performance is compared in Table 3. Relative to the global OLS model, which produced an adjusted $R^{2}$ of 0.682 and an AICc of 606.497, MGWR improved the fit substantially, raising the adjusted $R^{2}$ to 0.820 and reducing the AICc to 492.296. The residual diagnostics point in the same direction. For the OLS model, Moran’s *I* remained positive at 0.330 ( p < 0.01). After MGWR was fitted, the corresponding value dropped to 0.024 with a p -value of 0.296, indicating that residual spatial autocorrelation was no longer statistically significant (Table 3). The variance inflation factors ranged from 1.080 to 4.760, suggesting no clear sign of problematic multicollinearity. GWR showed slightly higher $R^{2}$ and adjusted $R^{2}$ , but it also used more effective parameters. MGWR had a lower trace(S) and the lowest AICc, indicating a more parsimonious fit. It was selected because the analysis focused on variable-specific spatial scales, not only on maximizing in-sample fit. Taken together, these results indicate that MGWR is better suited to capturing the multiscale spatial processes through which urban conditions shape MHACI, and thus provides a stronger basis for understanding uneven multidimensional access to healthcare across Shanghai.

**Table 3:** Model comparison and diagnostic statistics.

Diagnostic metrics	OLS	GWR	MGWR
$R^{2}$	0.687	0.879	0.856
Adjusted $R^{2}$	0.682	0.836	0.820
Residual sum of squares (RSS)	110.179	42.489	50.815
Parameters (OLS) / trace(S)	7	92.945	68.765
Akaike Information Criterion (Anick)	606.497	511.960	492.296
Residual Moran’s *I* ( p -value)	0.330 ( p < 0.01)	—	0.024 ( p = 0.296, n.s.)

Residual Moran’s *I* was calculated using K-Nearest Neighbors (kind, *k* = 8) row-standardized weights with 999 permutations (two-sided). n.s. = not significant at the 5% level. Global Moran’s I for MHACI is 0.827 ( p < 0.01).

The MGWR results indicate that urban factors do not operate over a common spatial range in relation to MHACI. As reported in Table 4, transport infrastructure has a near-global bandwidth of 350, and its coefficient range remains narrow, from 0.106 to 0.120. Child share, population density, and aging share operate at intermediate scales, with bandwidths of 143, 90, and 76, respectively, indicating that their coefficients vary more clearly across the city. Per capita GDP and local healthcare capacity have even smaller bandwidths, 48 and 50, suggesting more localized associations and stronger spatial non-stationarity. The following analysis combines these local coefficients with their significance patterns to show how urban conditions shape multidimensional healthcare access unevenly across Shanghai.

**Table 4:** Parameter estimates and bandwidth characteristics of the OLS and MGWR models.

Variables	OLS		MGWR				
	β	VIF	BW	Mean β	S.D.	Min	Max
Intercept	0.000	—	53	0.211	0.274	−0.224	0.823
Aging share	0.024	1.830	76	0.480	0.228	0.132	0.933
Child share	−0.281***	2.000	143	−0.100	0.092	−0.267	0.096
Per capita GDP	0.293***	1.590	48	0.365	0.225	0.012	0.866
Transport infrastructure	0.234***	4.760	350	0.109	0.003	0.106	0.120
Population density	0.395***	4.430	90	−0.298	0.359	−0.780	0.379
Local healthcare capacity	0.093***	1.080	50	0.064	0.136	−0.136	0.558

***denote p < 0.01; BW is the AICc-selected adaptive bisquare bandwidth ( N = 352).

#### 4.3.1 Demand-side: aging and child shares

The MGWR results show that Aging share operates at a smaller spatial scale than Child share. Its optimal bandwidth is 76, compared with 143 for Child share (Table 4), indicating sharper place-to-place variation in the effect of aging share.

**Figure 4:**
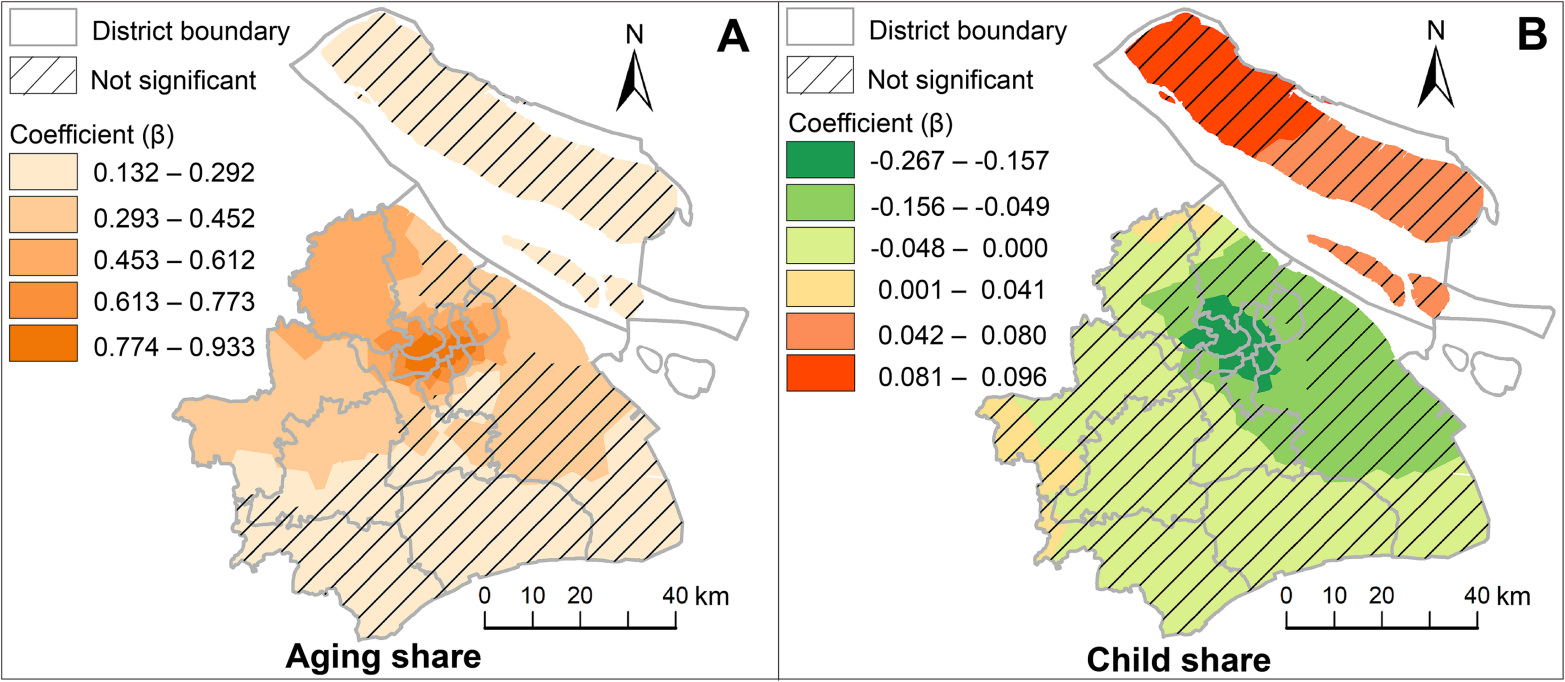
**(A)** Local coefficients of aging share estimated by MGWR, classified into five equal-interval classes; **(B)** local coefficients of child share estimated by MGWR, classified by separating negative and positive coefficients at zero and applying Jenks natural breaks within each range. Hatched areas indicate effects that are not significant at the 95% level based on the adjusted t -value criterion.

Across Shanghai, Aging share has a positive local association with MHACI. The mean coefficient is 0.480, with values ranging from 0.132 to 0.933 (Table 4). Stronger positive effects are concentrated in the urban core and nearby areas, then weaken toward the periphery. High-value clusters appear in central Shanghai and extend to the northwestern inner suburbs, whereas lower values are found mainly in the southern suburban zone and the island areas (Figure 4A). The significance pattern is broadly consistent with this distribution. In the urban core and neighboring units with medium to high coefficients, aging share shows a significant positive local effect on MHACI. In the southern suburbs and island areas, the local effects are not statistically significant, as indicated by the hatched zones in Figure 4A.

The local association between Child share and MHACI is more mixed. As reported in Table 4, the coefficients span both sides of zero, ranging from −0.267 to 0.096, with a mean of −0.100. Negative effects occupy most of the map. They are strongest in the urban core and extend northward and southward from the center (Figure 4B). Toward the west and southwest, the negative coefficients weaken and approach zero. Positive effects appear only in limited areas, mostly in the islands and along a few urban boundary zones. The significance pattern is more concentrated than that of Aging share. Statistically significant effects appear only in the central city and in the belt extending to its north and south, where the coefficients remain negative. Elsewhere, the local effects are not significant, as shown by the hatched areas in Figure 4B. Overall, the negative association between Child share and MHACI is concentrated in the urban core, while most peripheral areas show no significant local effect.

These demand-side patterns suggest that demographic need is shaped by local service contexts. In central areas, dense hospital networks may make aging-related demand easier to translate into realized access. The negative association for child share in the core points to a more constrained choice environment for pediatric care.

#### 4.3.2 Enabling resources: per capita GDP and transport infrastructure

The two enabling-resource variables operate at very different spatial scales in the MGWR model (Table 4). Per capita GDP has an optimal bandwidth of 48, indicating a highly localized association with MHACI. Transport infrastructure, by contrast, has a bandwidth of 350, suggesting a much more spatially stable pattern.

**Figure 5:**
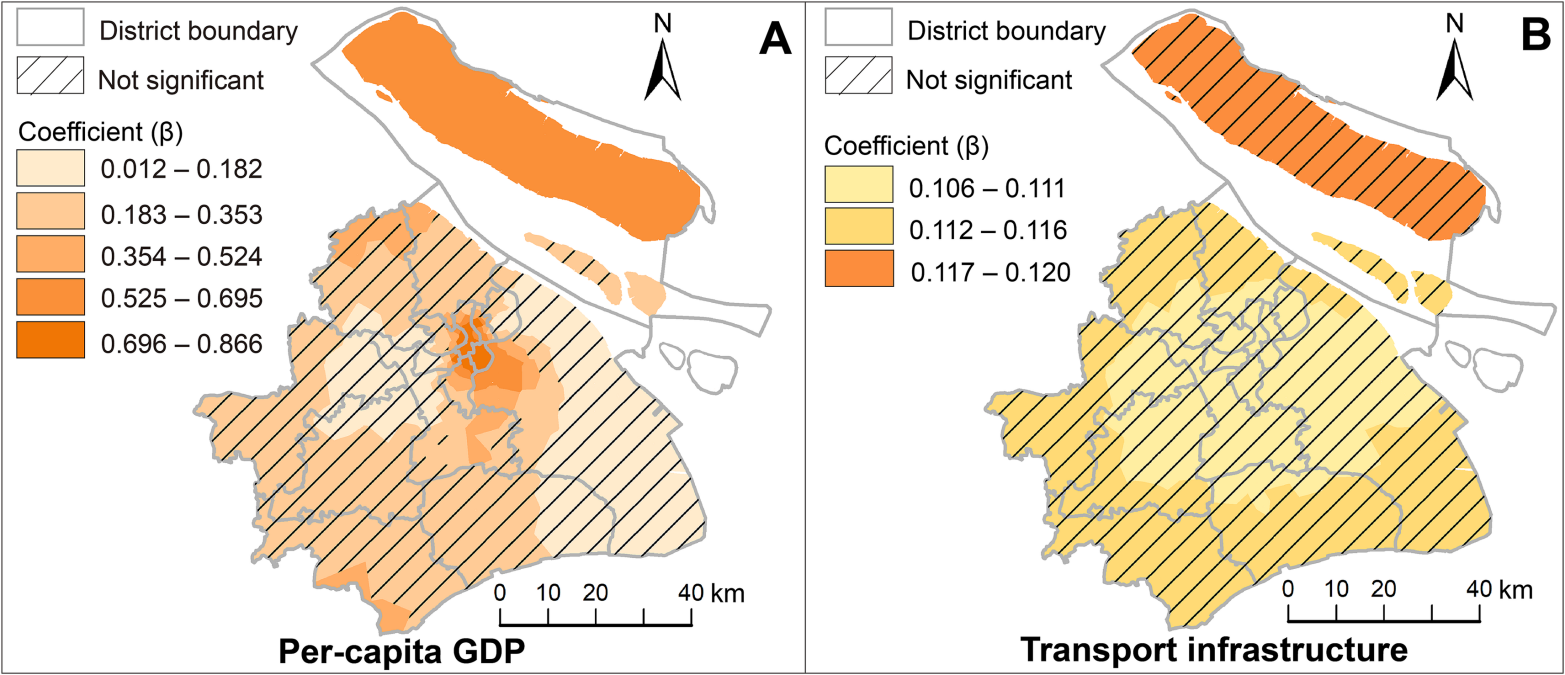
**(A)** Local coefficients of per capita GDP estimated by MGWR, classified into five equal-interval classes; **(B)** local coefficients of transport infrastructure estimated by MGWR, classified into three equal-interval classes. Hatched areas indicate effects that are not significant at the 95% level based on the adjusted t -value criterion.

Per capita GDP shows a positive local association with MHACI across the study area. Coefficients range from 0.012 to 0.866, with a mean of 0.365, but their strength varies sharply across space. Stronger effects are concentrated in two areas. One is the urban core and the adjoining southern area, where relatively high coefficients form a continuous cluster. The other is the island areas, where coefficients are both distinct from surrounding units and exceptionally high (Figure 5A). The significance pattern is more limited. Outside these two high-value zones, local effects are not statistically significant, as shown by the hatched areas in Figure 5A. In other words, the contribution of economic capacity to MHACI is concentrated in the urban core and a small number of high-resource areas.

Transport infrastructure showed a different pattern. Its bandwidth was nearly global, and its coefficient range was narrow, from 0.106 to 0.120. After the adjusted t value criterion was applied, the local coefficients were not statistically significant across the study area, as shown by the hatched areas in Figure 5B. For this reason, the coefficient sign and magnitude are not interpreted as evidence of a substantive local effect.

The localized effect of per capita GDP indicates that economic capacity did not operate evenly across the city. Its role was stronger where access to high-quality services was already more feasible, or where travel barriers made healthcare seeking more costly. Transport infrastructure showed little local differentiation after adjustment, so it is treated here as a background condition rather than a strong local driver.

#### 4.3.3 Supply-side: population density and local healthcare capacity

The MGWR results show clear spatial non-stationarity in the local effects of both supply-side variables on MHACI. Population density has an optimal bandwidth of 90, whereas Local healthcare capacity has a smaller bandwidth of 50. Both therefore operate at relatively local scales, with the latter showing stronger local sensitivity.

**Figure 6:**
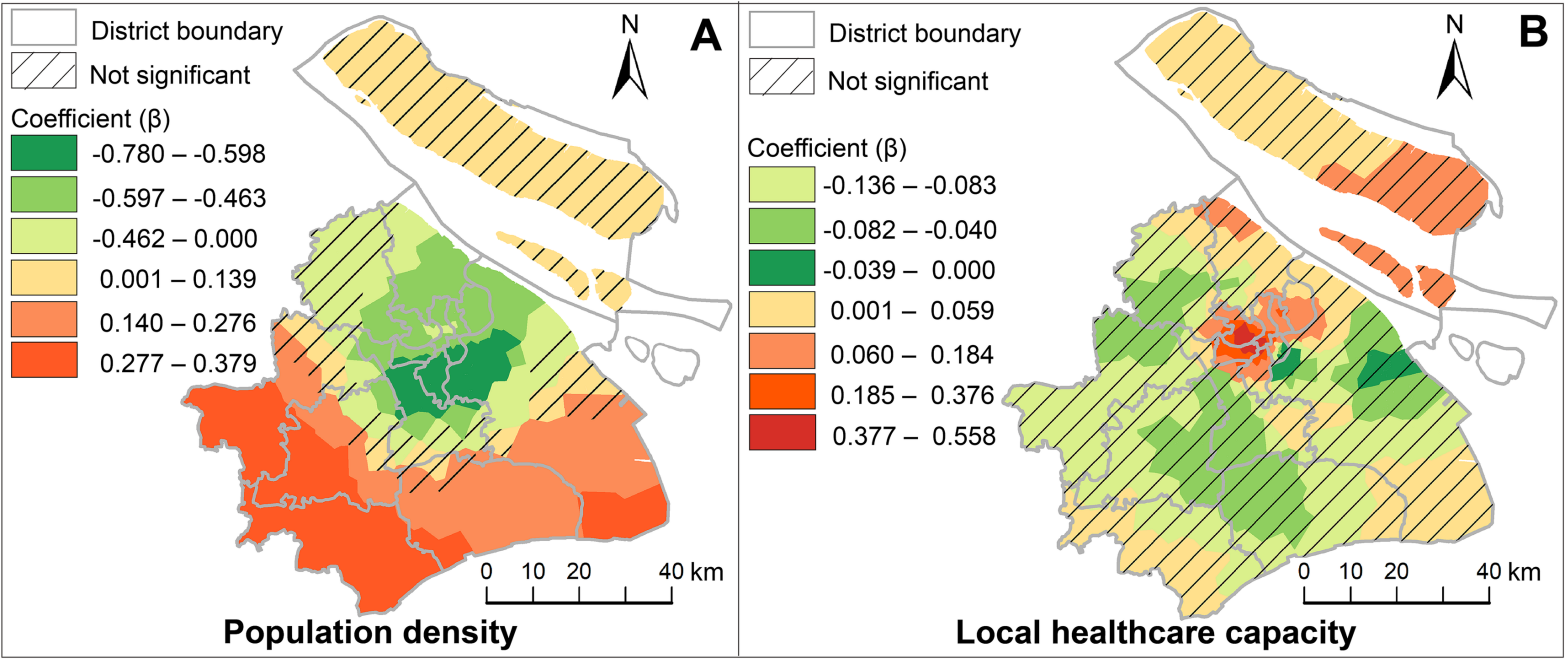
**(A)** Local coefficients of population density estimated by MGWR; **(B)** local coefficients of local healthcare capacity estimated by MGWR. Coefficients were visualized by separating negative and positive values at zero and applying Jenks natural breaks separately within each range. Hatched areas indicate effects that are not significant at the 95% level based on the adjusted t -value criterion.

The local effect of population density on MHACI is not uniform across Shanghai. As shown in Figure 6A, the coefficients cross zero, ranging from −0.780 to 0.379, with an average of −0.298. In the urban core and adjoining inner-city districts, its local association with MHACI is negative. Moving outward, these negative coefficients weaken and gradually shift toward positive values. In the southern and southwestern suburban areas, the local effect becomes positive and forms a continuous spatial zone. Except for the island areas and the transition zones between positive and negative coefficients, most local effects remain statistically significant, as indicated by the non-hatched areas in Figure 6A. Taken together, the pattern suggests that higher population concentration is associated with greater access pressure in the urban core, but with more favorable access conditions in peripheral areas.

Local healthcare capacity also spans positive and negative coefficients, ranging from −0.136 to 0.558. Its spatial pattern is more confined. Positive effects are concentrated mainly in the island areas, the urban core, and the belt extending to the north-east of the center, as shown in Figure 6B. Within this pattern, statistically significant positive effects appear only in the urban core and the northeastern extension. Elsewhere, coefficients fall into weakly positive or negative ranges, and most of these areas are covered by broad zones of non-significance. At the demand-unit scale, significant positive effects of Local healthcare capacity are therefore concentrated in a limited set of areas rather than distributed citywide.

The population density pattern points to two different urban contexts. In the core, density appears together with stronger pressure on concentrated services. In peripheral areas, it may provide a larger demand base for local provision. Local healthcare capacity also shows a selective effect, with stronger associations where facilities are embedded in denser hospital networks.

## 5 Discussion

### 5.1 Spatiotemporal mismatch and the reshaping of healthcare choice

Within Shanghai’s healthcare-seeking flow network, a large share of cross-district movement is directed toward the urban core. This pattern calls into question the territorially bounded assumptions that still underlie many conventional accessibility assessments (51). In a tiered hospital system, healthcare choice is not simply a matter of going to the nearest provider. It is shaped by hospital hierarchy, perceived quality, and the mobility routines built into everyday urban life. For this reason, observed healthcare-seeking flows offer a more credible basis for understanding how residents actually seek and use care across the city.

The spatial pattern of MHACI follows this logic closely. Higher values are concentrated in the central city, while lower values appear more often in transition zones, peripheral new towns, and island areas. This is not only a distance effect. It reflects the combined influence of hospital hierarchy, transport conditions, and the uneven location of high-quality hospitals. In central areas, lower spatial impedance and denser access to tertiary hospitals give residents both wider choice and stronger links to high-quality care. Conditions are different on the urban edge. There, feasible options are fewer, and seeking care across districts often brings extra time costs, longer trips, and greater uncertainty. What emerges is a broader gap in realized multidimensional healthcare access, not just a simple difference in geographic proximity. Studies in Chinese metropolitan areas have also reported clear spatial disparities in hospital accessibility (52). Patient mobility research further shows that healthcare flows can reshape accessibility and equality beyond fixed administrative boundaries (53). Our findings are consistent with this evidence, but add intra-urban flow evidence from Shanghai. Inequality is not only located in hospital distribution. It is also expressed through cross-district healthcare seeking and uneven connections to higher-tier hospitals.

This divide points to a broader problem of urban health inequality. Populations in peripheral areas, especially those already located at social or spatial margins, are more likely to encounter healthcare vulnerability. Obtaining high-quality care often involves greater travel burdens, time costs, and economic pressure. The sharp mismatch between dynamic healthcare demand and a relatively fixed distribution of healthcare resources therefore adds further evidence of the spatial mismatch of healthcare provision in megacities (54). This matters for how tiered healthcare provision works in practice. Formal coverage or nominal service presence does not automatically translate into effective access if residents remain weakly connected to appropriate levels of care under everyday mobility constraints. In this sense, the sustainability of healthcare access depends not only on where resources are located, but also on whether the service system can respond to observed healthcare-seeking flows in ways that improve supply–demand matching and support the service-side implementation of medical security.

### 5.2 Demand-side heterogeneity in population structure

Aging share has a positive and statistically significant association with MHACI in the urban core. In central districts, denser medical resources, better transport conditions, and stronger links to tertiary hospitals make it easier for aging-related healthcare needs to be translated into realized multidimensional access (55). This association becomes much less stable in the periphery. Across many outer areas, the coefficient weakens and loses statistical significance, suggesting that where local supply is thinner and connections to higher-tier care are weaker, aging-related demand is less likely to produce observable access advantages.

A different pattern appears for child share. In the urban core and the north–south belt extending from it, a higher child share is associated with lower MHACI. One likely reason is that parents tend to seek care from public general hospitals with stronger pediatric capacity, which narrows the set of providers they are willing to consider (56, 57). Another is the concentration of both healthcare resources and healthcare-seeking flows in central districts, where pediatric care is often sought under crowded conditions (58). For families accompanying children, this means tighter competition, less flexibility in provider choice, and greater time costs. Recent evidence from Shanghai also shows that caregivers’ choices for young children are shaped by the available healthcare system and access to professional guidance (59). Trust in primary care may further affect whether families use local providers, as primary care contracts have been associated with higher trust in general practitioners in China (60). The negative association is far less stable in peripheral areas, where limited supply appears to constrain access more generally. Demand-side heterogeneity, therefore, does not translate into healthcare access in any uniform way; its effects are filtered by the urban conditions under which care is actually sought and obtained.

### 5.3 Threshold effects and the foundational role of enabling resources

Per capita GDP shows a positive and statistically significant association with MHACI in the urban core. In these resource-dense and highly competitive districts, economic capacity does not create access on its own. Instead, it helps residents convert existing urban advantages into stronger links to high-quality hospitals within the healthcare-seeking network (61). A similarly strong positive effect also appears in the island areas, despite their geographic isolation. There, economic capacity appears to serve a different function by helping residents overcome cross-river barriers and seek high-quality care in the central city, thereby improving both connectivity to high-quality resources and the diversity of healthcare choices. By contrast, large parts of the urban periphery remain non-significant. This implies that where healthcare resources are broadly scarce, economic gains alone are not enough to produce meaningful differences in healthcare access capability. Peripheral areas must first cross a basic threshold of service provision before higher income can be translated into stronger access.

Transport infrastructure requires a cautious interpretation. Its local coefficients were not statistically significant, and spatial variation was limited. The result does not support interpreting transport infrastructure as a differentiated local driver of MHACI in this model. It is better read as a background condition in Shanghai, where basic transport connectivity is already widely developed and where hospital hierarchy and healthcare-seeking preferences may explain more of the observed spatial contrast.

### 5.4 Crowding penalty and agglomeration benefits under supply pressure

The local effect of population density on MHACI shows a clear spatial reversal. In the southern and southwestern suburban areas, higher density is associated with higher MHACI. This suggests that population growth in these areas has been accompanied by parallel expansion in public services and healthcare provision, allowing agglomeration benefits to emerge (62). The pattern is different in the urban core. Under already saturated conditions, additional population pressure tends to dilute per-capita medical resources and intensify competition for care, producing a crowding penalty and a significantly negative association with MHACI (63). Across most of the mainland city, this shift from agglomeration benefit to crowding penalty points to a persistent tension between demographic concentration and healthcare carrying capacity.

The effect of local healthcare capacity is also highly context-dependent. Its positive and statistically significant association with MHACI is concentrated in the central city. In this resource-rich setting, local improvements in healthcare capacity can strengthen both physical proximities to care and connectivity within the broader hospital system, which in turn raises multidimensional healthcare access for inner-city residents. The same pattern does not extend to much of the periphery. Where high-tier hospitals remain sparse and network connections are weaker, incremental improvements in basic facility capacity do not easily translate into wider system advantages. Proximity alone is therefore not enough. Under a tiered hospital system, the sustainability of urban healthcare access depends not only on how much capacity is added, but also on where it is located and how well it is integrated into the wider service network.

### 5.5 Limitations and future directions

The mobile signaling data cover only 1 month, which limits the ability to capture follow-up visits and seasonal variation. Trajectory inference from mobile signaling data also involves uncertainty, as spatial positioning errors, hourly temporal resolution, and complex activities around hospitals may affect the distinction between healthcare seeking and other hospital-related presence. Short visits, accompanying behavior, work-related stays, and boundary locations may therefore introduce residual misclassification. Longer time series would help test the robustness of the findings.

The results may also still be affected by the modifiable areal unit problem (MAUP). Voronoi units reduce reliance on administrative boundaries and give each origin location a unique assignment, but they cannot remove scale and zoning effects. Large peripheral units may contain internal heterogeneity, so future work should compare Voronoi units with street or township units and regular grids. Further progress will also depend on richer data integration. Combining electronic medical records and health insurance claims could help distinguish healthcare-seeking behavior across disease types, while information on healthcare preferences and patient satisfaction would shed more light on the micro-level mechanisms shaping multidimensional healthcare access in cities.

## 6 Conclusion and recommendations

### 6.1 Conclusion

Using Shanghai as the case, this study reconstructed healthcare-seeking flows from mobile signaling data and built an urban healthcare-seeking flow network. On that basis, it developed the Multidimensional Healthcare Access Capability Index (MHACI) from three dimensions: spatial accessibility, healthcare-seeking choice diversity, and connectivity to high-quality healthcare resources. The results show a clear core-periphery gradient, with higher MHACI values in the urban core and lower values in peripheral areas. The large share of cross-district healthcare seeking and the strong inward concentration of flows also suggest that healthcare use in a megacity cannot be adequately explained by the conventional assumption of nearby care seeking.

Multiscale geographically weighted regression further shows that the factors associated with MHACI operate at different spatial scales. Aging share is positively associated with MHACI in the urban core, whereas child share is linked to a competitive constraint in central areas. Transport infrastructure did not show significant local differentiation, while per capita GDP and local healthcare capacity worked at more localized scales. Population density also shifts from a crowding burden in the urban core to agglomeration benefits in peripheral areas.

These findings indicate that the spatial inequities of multidimensional healthcare access are closely related to the mismatch between relatively fixed healthcare provision and dynamic healthcare-seeking demand. Mobile signaling data help reveal how tiered hospital systems are actually used and where healthcare supply–demand matching becomes strained in practice. For megacities such as Shanghai, improving health equity requires not only broader service provision, but also better alignment between healthcare resources and observed healthcare-seeking flows. This has direct implications for tiered healthcare provision and the service-side implementation of medical security.

### 6.2 Policy implications

Policy responses should follow the spatial pattern of multidimensional healthcare access revealed by the healthcare-seeking flow network, rather than the nominal distribution of facilities alone. In the urban core, the main issue is the concentration of both hospitals and patient flows. Community hospitals therefore need to absorb more chronic disease management and routine care, while tertiary hospitals should strengthen outpatient triage and patient diversion. In densely populated central districts, community-level pediatric services should be made more specific and visible. Community health centers could provide standardized pediatric clinics for common childhood illnesses, child health management, vaccination follow-up, and routine consultation. Tertiary hospitals and community facilities should also build tighter referral links, including triage rules, appointment channels, specialist support, and post-visit follow-up.

In peri-urban areas and new towns, the priority is not only to add facilities, but to improve how local services connect to the wider hospital system. This requires stronger medical alliances, clearer referral pathways, and more effective tiered diagnosis and treatment. Regional medical centers can help link secondary hospitals, community hospitals, and specialist services so that more demand is managed locally rather than continuously drawn to the urban core.

Outer suburban areas and island zones face a more basic access problem. Long travel times, cross-river barriers, and weak links to high-quality hospitals still limit healthcare choice. In these areas, the first task is to strengthen the safety-net role of primary care. Teleconsultation, stable referral channels, and family physician follow-up are especially important for older adults and other groups with continuing care needs.

These findings also show the practical value of mobile signaling data and other mobility-sensitive big data. When used in aggregated and privacy-protected form, such data can help track healthcare-seeking flows, identify persistent supply–demand mismatches, and support more targeted referral guidance. For megacities, this kind of evidence can also inform medical alliance planning and the service-side improvement of medical security.

## Data Availability

The raw mobile signaling data analyzed in this study are not publicly available due to privacy protection and data use restrictions. Aggregated and anonymized derived data supporting the findings may be available from the corresponding author upon reasonable request, where permitted by the data provider and relevant regulations.
