# Supplementary material for: Mobile signaling big data, healthcare-seeking flows, and multidimensional healthcare access in Shanghai: implications for healthcare supply–demand matching and medical security governance
Source: Front Public Health. 2026 Jun 2;14:1841112. doi: 10.3389/fpubh.2026.1841112 (PMC13269089; doi:10.3389/fpubh.2026.1841112)
Supplement: Supplementary file 1 [file Table_1.DOCX]

Supplementary Table S1. Data sources and harmonization

| Data type | Main source | Time | Original scale | Harmonized analytical use |
| --- | --- | --- | --- | --- |
| Hospital roster and level | Shanghai Municipal Health Commission official directories | 2019 roster | Hospital list | Selection of 352 hospitals and level classification |
| Hospital point location | Amap POI data and official hospital rosters | Matched to 2019 roster | Hospital point locations | Geocoding and demand unit construction |
| Hospital resource attributes | Shanghai Health Statistics, hospital websites, and offline verification | 2019 annual data | Hospital | Local healthcare capacity and tier weighted accessibility |
| Population and socioeconomic data | Population census, Shanghai Statistical Yearbook, and district statistical bulletins | 2019 annual cross section | Statistical areas | Demand unit level covariates |
| Transport infrastructure | Baidu Maps Open Platform | Reference for the 2019 study period | Roads, metro stations, and bus stops | Demand unit level transport index |
| Administrative boundaries | National Catalogue Service for Geographic Information Resources | Reference for the 2019 study period | Municipal and district polygons | Spatial matching and mapping |
| Mobile signaling records | Major telecommunications operator in Shanghai | March 2019 | Hourly location records | Monthly healthcare seeking OD flows |
